# Caring From Afar: Long-Distance Caregivers’ Use of Supportive Services for Themselves

**DOI:** 10.1093/geroni/igab046.1368

**Published:** 2021-12-17

**Authors:** Verena Cimarolli, Molly Wylie, Jillian Minahan Zucchetto, Francesca Falzarano, Amy Horowitz

**Affiliations:** 1 LeadingAge, Washington, District of Columbia, United States; 2 University of Massachusetts Boston, University of Massachusetts boston, Massachusetts, United States; 3 Fordham University, Psychology Department, Bronx, New York, United States; 4 Weill Cornell Medicine, Douglaston, New York, United States; 5 Fordham University, Graduate School of Social Service, New York, New York, United States

## Abstract

Although long distance caregivers (LDCs) are starting to be recognized as a subgroup of care partners experiencing unique challenges and stresses, it is unknown 1) what types of supportive services LDCs use for themselves and 2) what factors are associated with supportive service use in this understudied caregiving population. In our sample of 304 LDCs (Mage=56.9), the most frequently utilized service was video phone/webcam systems to monitor the care recipient (CR). Guided by Andersen’s Model of Health Care Utilization and using multiple hierarchical regression analysis, younger age of the LDC (a predisposing factor) and need-related characteristics (greater caregiving burden and depressive symptoms, more time spent helping the CR, and worse CR functional status) were associated with greater use of supportive services. Enabling factors were not associated with service use. These study findings can help inform how to engage LDCs in supportive service utilization.

